# PRRSV Induces HMGB1 Phosphorylation at Threonine-51 Residue to Enhance Its Secretion

**DOI:** 10.3390/v14051002

**Published:** 2022-05-08

**Authors:** Rong Wang, Jingyi Zhang, Yu Fu, Linying Jia, Yali Zhang, Liang Bai, Weirong Wang, Daxin Cheng, Enqi Liu

**Affiliations:** 1 Laboratory Animal Center, Xi’an Jiaotong University, Xi’an 710061, China; angelzjy@stu.xjtu.edu.cn (J.Z.); fuy2017@lzu.edu.cn (Y.F.); jialinying95202@stu.xjtu.edu.cn (L.J.); zhangyali2015@stu.xjtu.edu.cn (Y.Z.); bailiang0922@mail.xjtu.edu.cn (L.B.); szb2013072@mail.xjtu.edu.cn (W.W.); chengdaxinxjtu@126.com (D.C.); liuenqi@xjtu.edu.cn (E.L.); 2 Department of Laboratory Animal Science, School of Basic Medical Sciences, Xi’an Jiaotong University, Xi’an 710061, China

**Keywords:** porcine reproductive and respiratory syndrome virus, PRRSV, high-mobility group box 1 (HMGB1), HMGB1 secretion, ribosomal protein S3 (RPS3)

## Abstract

Porcine reproductive and respiratory syndrome virus (PRRSV) induces secretion of high mobility group box 1 (HMGB1) to mediate inflammatory response that is involved in the pulmonary injury of infected pigs. Our previous study indicates that protein kinase C-delta (PKC-delta) is essential for HMGB1 secretion in PRRSV-infected cells. However, the underlying mechanism in HMGB1 secretion induced by PRRSV infection is still unclear. Here, we discovered that the phosphorylation level of HMGB1 in threonine residues increased in PRRSV-infected cells. A site-directed mutagenesis study showed that HMGB1 phosphorylation at threonine-51 was associated with HMGB1 secretion induced by PRRSV infection. Co-immunoprecipitation (co-IP) of HMGB1 failed to precipitate PKC-delta, but interestingly, mass spectrometry analysis of the HMGB1 co-IP product showed that PRRSV infection enhanced HMGB1 binding to ribosomal protein S3 (RPS3), which has various extra-ribosomal functions. The silencing of RPS3 by siRNA blocked HMGB1 secretion induced by PRRSV infection. Moreover, the phosphorylation of HMGB1 at threonine-51 was correlated with the interaction between HMGB1 and RPS3. In vivo, PRRSV infection also increased RPS3 levels and nuclear accumulation in pulmonary alveolar macrophages. These results demonstrate that PRRSV may induce HMGB1 phosphorylation at threonine-51 and increase its interaction with RPS3 to enhance HMGB1 secretion. This finding provides insights into the pathogenesis of PRRSV infection.

## 1. Introduction

Porcine reproductive and respiratory syndrome (PRRS) is a serious contagious swine disease, causing significant economic loss to the swine industry across the world. Inflammatory injuries are the typical pathological manifestations of PRRS, such as interstitial pneumonia, vasculitis, lymphadenopathy, myocarditis, and encephalitis [1]. The causative agent of the disease is the PRRS virus (PRRSV), an enveloped, positive-sense, and single-stranded RNA virus belonging to the *Arteriviridae* family in the order *Nidovirales* [2]. The PRRSV genome is approximately 15 kb in length and encodes over ten open reading frames (ORFs). ORFs 1a and 1b comprise 80% of the viral genome and encode nonstructural proteins, whereas ORFs 2 to 7 encode structural proteins [3,4]. Porcine pulmonary alveolar macrophages (PAMs) are the main target cells for PRRSV during the acute infection of pigs [1]. In vitro, the epithelium-derived monkey kidney cell line MARC-145 [5] and primary PAMs are usually used to propagate PRRSV.

High mobility group box 1 (HMGB1) is a multifunctional protein [6,7]. Normally, it locates in the nucleus and functions as a non-histone chromatin-binding protein that is involved in DNA organization and transcriptional regulation. However, in the activated monocytes and macrophages, HMGB1 can be secreted into the extracellular environment and acts as a proinflammatory mediator to promote and exacerbate the inflammation responses [8,9]. It is reported that PRRSV infection induces HMGB1 secretion in vitro and in vivo, which contributes to the upregulation of inflammatory cytokines, as well as intensive pulmonary inflammatory injury [10,11,12,13]. In vivo, the highly pathogenic PRRSV strain, HuN4, significantly induced the secretion of HMGB1 and inflammatory cytokines (such as IL-1β, IL-6) into the bronchoalveolar lavage fluid of piglets [10]. There was also a significant positive correlation between levels of serum HMGB1 and IL-6 expression [11]. Duan et al. reported that PRRSV infection triggered the translocation of HMGB1 from the nucleus to the extracellular milieu, while HMGB1 promoted PRRSV-induced NF-κB activation and the subsequent expression of inflammatory cytokines through the receptors RAGE, TLR2 and TLR4 [13]. We also discovered that PRRSV induced HMGB1 nuclear translocation and secretion and that PRRSV ORF2b and ORF5a proteins were responsible for the induction [12].

Since lacking a secretory signal peptide, HMGB1 cannot be secreted via the classical endoplasmic reticulum–Golgi secretory pathway. Instead, HMGB1 secretion is regulated by a post-translational modification that occurred on its nuclear localization sequence (NLS), such as hyperacetylation and phosphorylation [14,15,16,17]. It is reported that activated JAK/STAT signaling mediates HMGB1 acetylation [18], while protein kinase C (PKC), a family of proteins with serine/threonine kinase activity, phosphorylate HMGB1 [19,20,21]. Our previous study shows that PKC-delta (PKCδ), a member of the PKC family, is essential for the HMGB1 subcellular relocation and secretion induced by PRRSV infection [12]. It implies that phosphorylation on HMGB1 might be closely related to its secretion in PRRSV-infected cells. However, the underlying mechanism has yet to be revealed.

In the present study, we found that HMGB1 phosphorylation at threonine-51 residue was crucial for the HMGB1 secretion induced by PRRSV infection. HMGB1 interacts with ribosomal protein S3 (RPS3), instead of PKCδ. RPS3 is a component of the 40S ribosome and has various extra-ribosomal functions, such as regulating DNA repair, apoptosis, and the innate immune response to bacterial infection [22,23]. We discovered that RPS3 was associated with HMGB1 secretion. Moreover, PRRSV induces RPS3 elevation and nuclear accumulation in PAMs. These findings contribute to our understanding of the pathogenesis of PRRSV infection.

## 2. Materials and Methods

### 2.1. Cells, Viruses, and Chemicals

MARC-145 cells [5] and HEK293T were obtained from ATCC and grown in Dulbecco’s Modified Eagle Medium (DMEM) supplemented with 10% fetal bovine serum (FBS). The PAM cells were harvested from PRRSV or PBS inoculated three-week-old PRRSV-negative pigs (*n* = 5 per group) in a previous study [10], which was approved by the Laboratory Animal Administration Committee of Xi’an Jiaotong University (Approval #: XJTULAC2016-318) according to the Guidelines for Animal Experimentation of Xi’an Jiaotong University and Guide for the Care and Use of Laboratory Animals published by the US National Institutes of Health (NIH Publication NO. 85-23, revised 2011). PRRSV HuN4 strain [24], a highly pathogenic PRRSV (HP-PRRSV) strain, was used to inoculate MARC-145 cells at a multiplicity of infection (MOI) of 1. Virus titers were determined in MARC-145 cells for the median tissue culture infectious dose (TCID50), as described previously [25].

Lipofectamine™ 2000 Transfection Reagent (Cat. No. 11668-027, Invitrogen, Waltham, MA, USA) and HiPerFect Transfection Reagent (Cat. No. 301704, QIAGEN, Hilden, Germany) were used to transfect plasmid DNA and siRNA, respectively, into the cells according to the manufacturer’s instructions.

### 2.2. Plasmids and siRNA

ORF2b and ORF5a of HuN4 were cloned into the pCDNA3-VenusC1 vector, and HMGB1 was cloned into the PCAGEN-HA vector, as described [12,26]. The resulting recombinant plasmids were confirmed by restriction enzyme digestion and DNA sequencing. Their overexpression in transfected cells was confirmed by fluorescence microscopy and immunoblotting.

The mutant plasmids of HMGB1, namely T51A and T51E, were constructed to substitute threonine-51 (T51) in the NLS regions of HMGB1 with alanine (A) or glutamate (E), as indicated. All the mutant plasmids were constructed by sequential PCR as described previously [25], and the sequences were confirmed by direct sequencing analysis. Primers used in this study were listed in Table 1.

Sequences of siRNA targeting RPS3 (siRPS3) and non-targeting control siRNA (NC) are 5′-GCGGAGACCCUGUUAACUATT-3′ [27] and 5′-UUCUCCGAACGUGUCACGUTT-3′, respectively. The siRNA duplexes were synthesized and purified by Sangon Biotech (Shanghai, China).

### 2.3. Co-Immunoprecipitation (co-IP)

Co-IP was conducted as previously described [25]. Cells were lysed with a lysis buffer (50 mM Tris, pH 7.4, 150 mM NaCl, 0.2 mM EDTA, 2 mM EGTA, 0.5% Igepal CA-630, 10% glycerol, 1 mM sodium vanadate) supplemented with a protease inhibitor (Cat. No. 4693132001, Roche, Basel, Switzerland) and a phosphatase inhibitor (Cat. No. 4906837001, Roche). The lysate was clarified by centrifugation at 14,000× *g* for 5 min at 4 °C. The supernatant was transferred to a new tube and incubated with antibodies against HMGB1 (Cat. No. ab190377, Abcam, Cambridge, UK) or RPS3 (Cat. No. ab140688, Abcam), followed by incubation with Dynabeads^TM^ protein G (Cat. No.10003D, Invitrogen). The final pellet from IP was subjected to elusion with the Laemmli sample buffer or 100 mM glycine solution, pH 3.0. The IP samples were subjected to immunoblotting with antibodies against phosphorylated serine (p-Ser) (Cat. No. ab9332, Abcam), phosphorylated threonine (p-Thr) (Cat. No. ab9337, Abcam), HMGB1 (Cat. No.ab79823, ab190377, Abcam), RPS3 and GFP (Cat. No. ab13970, Abcam), respectively.

### 2.4. Immunoblotting

Protein samples were separated by sodium dodecyl sulfate-polyacrylamide gel electrophoresis (SDS-PAGE) and analyzed by immunoblotting as described previously [25]. The separated proteins were transferred onto the PVDF membrane and probed with antibodies against p-Ser, p-Thr, HMGB1, RPS3, GFP, α-tubulin (Cat. No. ab15246, Abcam), and histone H2A (Cat. No. 7631, Cell Signaling Technology, Danvers, MA, USA). The antibodies bound on the membrane were detected with secondary antibodies conjugated with horseradish peroxidase (Cat. No. HS-101, HS-201, Transgen, Beijing, China), followed by revealing with a chemiluminescence substrate. The luminescence signal was recorded digitally by using a Chemi-Doc XRS imaging system (Bio-Rad, Hercules, CA, USA). Digital image acquisition and analysis were conducted using the Quantity One program (Bio-Rad).

### 2.5. Immunofluorescence Assay (IFA)

MARC-145 cells were seeded into wells of a culture plate with coverslips, incubated overnight, and then infected with PRRSV HuN4 at an MOI of 1. The cells were fixed with 2% paraformaldehyde at 24 h post-infection (hpi), followed by IFA with antibodies against HMGB1 and RPS3 as described [26]. The specific reactions were detected by the conjugated secondary antibodies: goat anti-mouse IgG (H&L) Cy3 (Cat. No. ab97035, Abcam) and anti-rabbit IgG (H&L), F(ab’)2 Fragment (Alexa Fluor^®^ 488 Conjugate) (Cat. No. 4412S, CST). The coverslips were mounted onto slides using DAPI Fluoromount-G (Cat. No. 0100, Southern Biotech, Birmingham, AL, USA). The fluorescence signal was observed under confocal fluorescence microscopy (Nikon C2), and images were taken with NIS-Elements version 4.0 (Nikon).

### 2.6. RNA Isolation and Real-Time PCR

Total RNA was isolated from MARC-145 cells with RNAiso Plus (Cat. No. 9108, Takara, Kusatsu, Japan) following the manufacturer’s instructions. Reverse transcription and real-time quantitative PCR (RT-qPCR) were conducted with a PrimeScript™ RT reagent Kit (Cat. No. RR047A, Takara) and SYBR Green Pro Taq HS (Cat. No. AG11701, Accurate Biology, Hunan, China) according to the manufacturers’ instructions, respectively. Primers used in this study are listed in Table 1. Transcripts of ribosomal protein L32 (RPL32) were amplified from the samples of MARC-145 and used to normalize the total amount of input RNA. Relative transcript levels were quantified by the 2^−ΔΔ*C*T^ (threshold cycle) method [28] and were shown as relative fold change in comparison with the level for the mock-treated control.

### 2.7. Subcellular Fractionation

The PAM cells, harvested from PRRSV HuN4 or PBS inoculated pigs in a previous study [10], were used for subcellular fractionation by using a Cell Fractionation kit (Cat. No. 9038, Cell Signaling Technology) following the manufacturer’s instructions [12]. The cytoplasmic and nuclear fractions were subjected to immunoblotting with antibodies against RPS3, α-tubulin and histone H2A.

### 2.8. Statistical Analysis

Data are expressed as mean ± standard error. A Student’s *t*-test (for comparison between the treatment group and control group) was used for the statistical analyses. GraphPad Prism 5 software was used. A two-tailed *p* value of less than 0.05 was considered significant.

## 3. Results

### 3.1. PRRSV Infection Induces HMGB1 Phosphorylation at Threonine Residues

**Figure 1:**
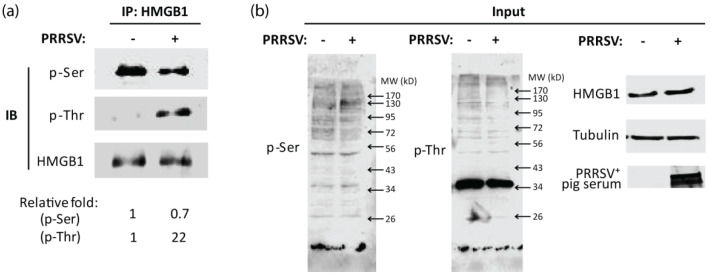
PRRSV infection induces HMGB1 phosphorylation at threonine residues. The MARC-145 cells were infected with PRRSV stain HuN4 at a multiplicity of infection (MOI) of one and 24 h post-infection (hpi), harvested for further analysis. (**a**) PRRSV infection increases the phosphorylation level of HMGB1 at threonine residues. Immunoprecipitation (IP) was done with HMGB1 antibodies, followed by immunoblotting (IB) with antibodies against phosphorylated serine (p-Ser), phosphorylated threonine (p-Thr), and HMGB1. Relative levels of p-Ser and p-Thr are shown as folds below the images after normalization with HMGB1 in densitometry analysis. (**b**) IB results for input whole cell lysate. Whole gel images for p-Ser and p-Thr IB are shown. PRRSV-positive (PRRSV^+^) pig serum was also used for detecting viral proteins.

It is known that serine (Ser) and threonine (Thr) are the main residues targeted by PKC. To determine which amino acid on NLS is critical for HMGB1 secretion in PRRSV-infected cells, we conducted HMGB1 IP and analyzed the phosphorylation levels of HMGB1 on Ser and Thr, with antibodies against p-Ser and p-Thr, respectively. Compared with mock-infected cells, PRRSV infected cells had 22-fold higher levels of p-Thr HMGB1 and 0.7-fold p-Ser HMGB1 (Figure 1a). In whole-cell lysates, PRRSV infection led to a minimal change in total proteins’ Ser or Thr phosphorylation (Figure 1b). The results indicate that PRRSV induces HMGB1 phosphorylation at the threonine residues.

### 3.2. HMGB1 Phosphorylation at Threonine-51 Is Associated with the HMGB1 Secretion Induced by PRRSV Infection

**Figure 2:**
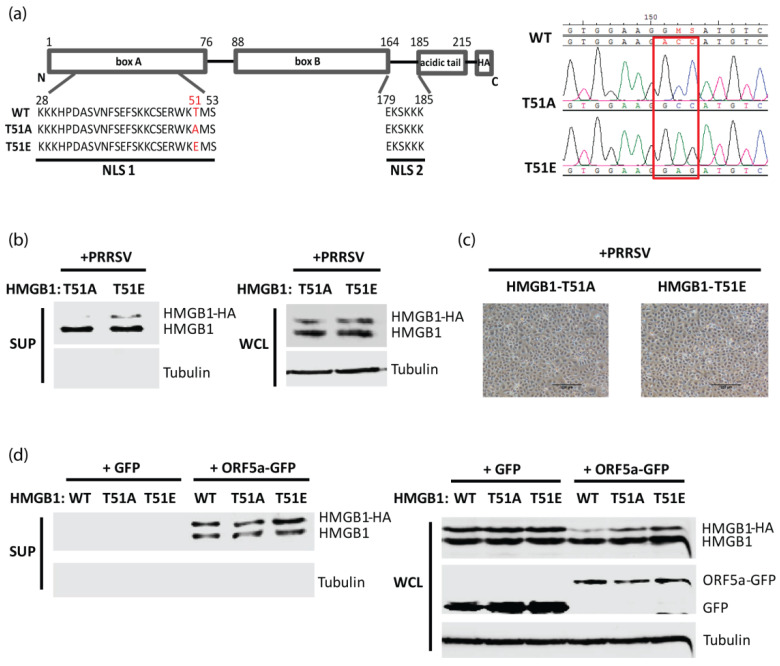
The threonine-51 residue (Thr-51) within the nuclear localization sequence (NLS) of HMGB1 is responsible for PRRSV-infection induced HMGB1 secretion. (**a**) Schematic illustration of HMGB1 NLS in wild type (WT) and mutant HMGB1 plasmids. T51A or T51E denotes mutant HMGB1 with a mutation of threonine-51 to alanine or glutamic acid, respectively. The mutated amino acids are written in red. The chromatogram on the right shows the sequencing results of T51A and T51E. The mutant nucleotide sites are shown in the red square. (**b**) HMGB1 phosphorylation at Thr-51 is related to the HMGB1 secretion induced by PRRSV infection in MARC-145 cells. MARC-145 cells were transfected with plasmids expressing HMGB1-T51A or HMGB1-T51E. At 24 h post-transfection, the cells were infected with HuN4 at an MOI of 1 and harvested 24 h later. The supernatant (SUP) and whole-cell lysate (WCL) were harvested for IB. Cell morphology before the harvesting is shown in (**c**). Bars in the images denote 250 μm. (**d**) HMGB1 phosphorylation at Thr-51 is related with PRRSV ORF5a-induced HMGB1 secretion. HEK293T cells were transfected with HMGB1 WT, T51A, or T51E plasmid, along with GFP or PRRSV ORF5a-GFP plasmid. Thirty hours later, the SUP and WCL were harvested for IB with antibodies against HMGB1, GFP, and tubulin.

On the NLS of HMGB1, there is only one threonine, which is located at residue 51 (Figure 2a). HMGB1 plasmids with point mutations were constructed to substitute threonine-51 (T51) with alanine (A) or glutamate (E). The two constructs, T51A and T51E, simulate an inactive (unphosphorylated) and an active (phosphorylated) state of HMGB1, respectively. DNA sequencing confirmed the point mutation in the mutant HMGB1 plasmids (Figure 2a). MARC-145 cells were transfected with T51A or T51E and then infected with PRRSV. The immunoblotting result showed that the endogenous HMGB1 levels in the supernatant of T51A and T51E transfected cells were similar (Figure 2b). However, the levels of secreted exogenous mutant HMGB1 that are HA-tagged (T51A and T51E) showed a difference: T51E secreted into the supernatant was more than the trace amount of T51A (Figure 2b). Cell morphology of T51A or T51E-transfected cells was similar (Figure 2c). Our previous study shows that PRRSV induces HMGB1 translocation and secretion and that PRRSV ORF2b and ORF5a proteins are responsible for the induction [12]. Therefore, we co-transfected HEK293T cells with ORF5a-GFP plasmid and one of the following plasmids: wild-type (WT) HMGB1, T51A, or T51E. The result showed that compared with the GFP empty vector, ORF5a-GFP induced endogenous HMGB1 and mutant HMGB1-HA secretion into the supernatant (Figure 2d). Moreover, T51E showed higher secretion levels than T51A in the transfected cells (Figure 2d). In whole-cell lysates, endogenous HMGB1 expression levels were similar among different transfection groups (Figure 2d). However, ORF5a-GFP strongly influenced WT HMGB1-HA plasmid expression, which might be the reason for the HMGB1 WT secretion of less than T51E in the supernatant.

### 3.3. PRRSV Enhances HMGB1 Interaction with RPS3

**Figure 3:**
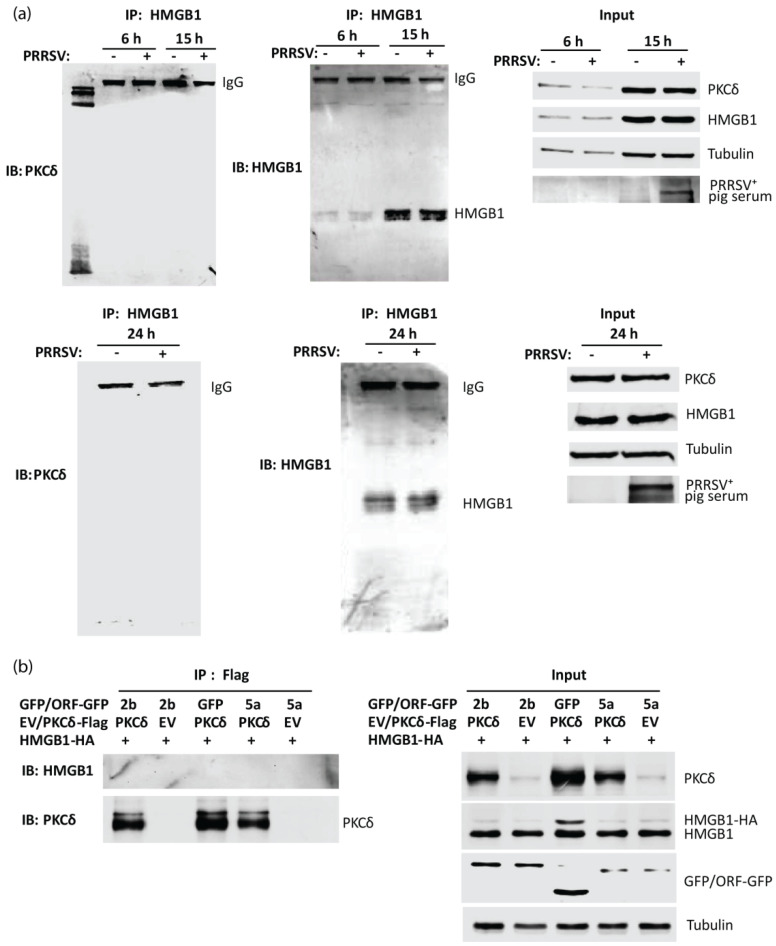
The interaction between PKCδ and HMGB1 is below the detection level. (**a**) HMGB1 IP fails to precipitate PKCδ in PRRSV-infected MARC-145 cells. The cells were infected with HuN4 at an MOI of 1 and were harvested for IP with HMGB1 antibody at 6, 15, and 24 hpi, followed by IB. The band of immunoglobulin G (IgG) from IP elusion is indicated. The input was included in IB. PRRSV^+^ pig serum was used for detecting PRRSV infection. (**b**) PKCδ IP fails to precipitate HMGB1 in HEK293T cells. The cells were co-transfected with HMGB1-HA, PKCδ-Flag, and PRRSV ORF2b or ORF5a plasmids. At 30 h after transfection, the cells were harvested for IP with Flag antibody, followed by IB with antibodies against HMGB1 and PKCδ. The input was included in IB.

Our previous study shows that PKCδ mediates the HMGB1 secretion induced by PRRSV infection [12]. Therefore, we wondered whether there was an interaction between HMGB1 and PKCδ. A Co-IP with antibodies against HMGB1 was conducted at 6, 15, and 24 hpi, followed by immunoblotting with antibody against PKCδ. The result showed the absence of PKCδ (Figure 3a). To confirm the observation, we co-transfected HEK293T cells with plasmids encoding ORF2b-GFP or ORF5a-GFP along with PKCδ-flag and HMGB1-HA, then conducted co-IP with antibody against Flag. The result of immunoblotting with antibodies against HMGB1 showed that there was no HMGB1 band visible (Figure 3b). These results suggest that the interaction between HMGB1 and PKCδ might be transient or beyond the detection by this co-IP assay.

To determine which proteins interact with HMGB1 in PRRSV-infected cells, we infected MARC-145 cells with HuN4 and at 24 hpi, harvested the cells for co-IP with antibody against HMGB1. Mass spectrometry (MS) was done to analyze the HMGB1 co-IP product. The result showed that in comparison with the mock-infected cells, three proteins exist only in the sample of the PRRSV-infected cells (Table 2). Among the three proteins, two were uncharacterized and the third one was RPS3, a component of the 40S small ribosomal subunit, involved in protein translation, DNA repair, transcription regulation, apoptosis, innate immune response, and so on [22,23].

**Figure 4:**
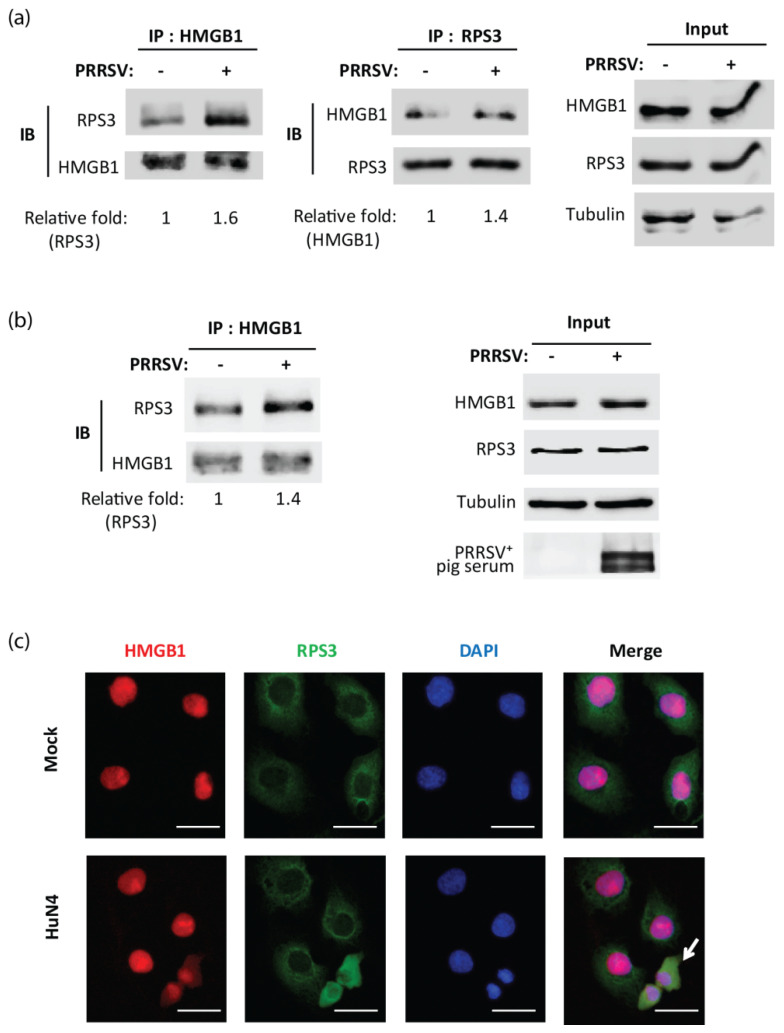
PRRSV increases HMGB1 binding to RPS3 in MARC-145 cells. MARC-145 cells were infected with HuN4 at an MOI of 1 for 6 h (**a**) and 24 h (**b**). The cells were harvested for IP with antibodies against HMGB1 or RPS3, followed by IB. Relative levels of RPS3 and HMGB1 are shown as folds below the images after normalization with HMGB1 or RPS3, respectively, in a densitometry analysis. The input was also included in IB. PRRSV^+^ pig serum was used for detecting PRRSV infection. (**c**) Immunofluorescence assay (IFA) shows the co-localization of HMGB1 and RPS3. MARC-145 cells were infected with HuN4 at an MOI of 1 and, 24 h later, were fixed for IFA with antibodies against HMGB1 (red) and RPS3 (green). DAPI staining of nuclear DNA is also shown (blue). Arrow points to the cell showing HMGB1-RPS3 co-localization. Bar in images denotes 25 μm.

To confirm whether HMGB1 interacts with RPS3, we conducted co-IP with antibodies against HMGB1 and RPS3 for mock- or HuN4-infected MARC-145 cells at 6 hpi. The immunoblotting result showed that PRRSV infection enhanced the interaction between HMGB1 and RPS3 (Figure 4a). When the infection was prolonged to 24 h, the interaction between HMGB1 and RPS3 still existed (Figure 4b). IFA results also showed the colocalization of HMGB1 and RPS3 in PRRSV-infected cells (Figure 4c).

### 3.4. RPS3 Is Involved in HMGB1 Secretion in PRRSV-Infected Cells

**Figure 5:**
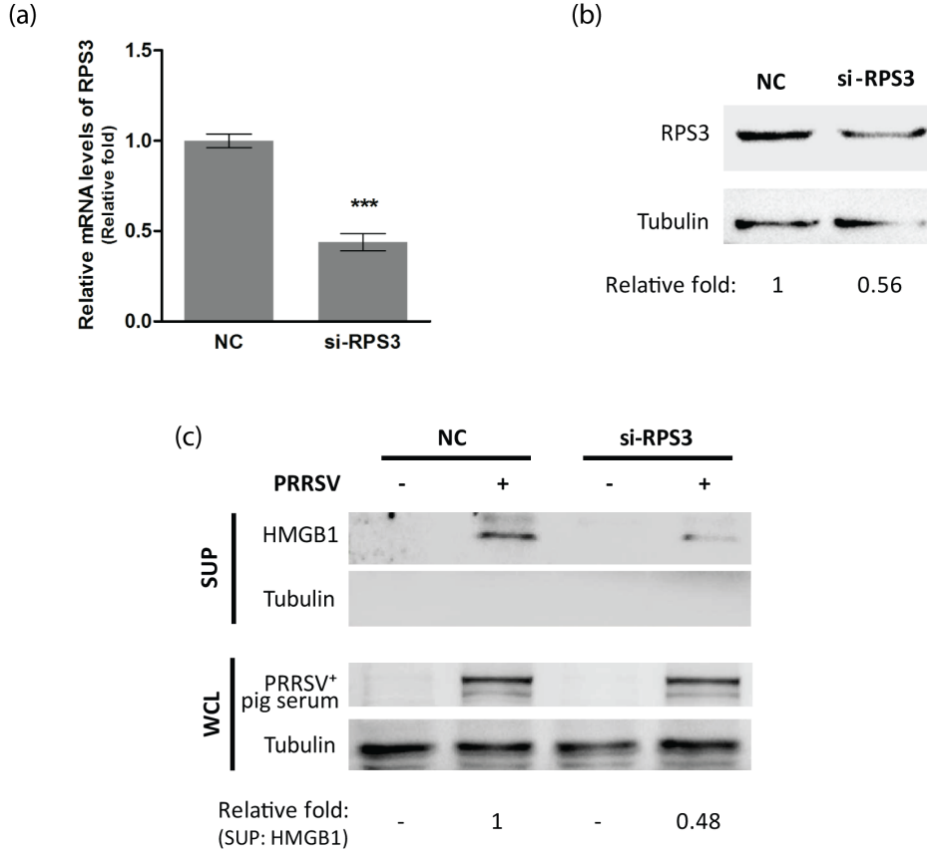
Silencing of RPS3 diminishes the HMGB1 secretion induced by PRRSV infection. (a&b) RPS3 mRNA and protein levels in cells transfected with siRNA control (NC) or siRNA against RPS3 (si-RPS3). MARC-145 cells were transfected with NC or si-RPS3 (100 nM) and, 48 h later, were harvested for RT-qPCR (**a**) and IB (**b**). Significant differences are denoted by “***” for *p* < 0.001. (**c**) Silencing of RPS3 reduces the HMGB1 secretion induced by PRRSV infection. MARC-145 cells treated with NC or si-RPS3 for 24 h were infected with HuN4 at an MOI of 1. The SUP and WCL were harvested at 24 hpi for IB with antibodies against HMGB1 and tubulin. PRRSV^+^ pig serum was also used in IB for detecting PRRSV infection. Relative levels of HMGB1 in SUP are shown as folds below the images.

To test whether RPS3 was associated with the HMGB1 secretion induced by PRRSV infection, we conducted RNAi-mediated silencing of RPS3 with siRNA. The knockdown effect of si-RPS3 on RPS3 expression in transcription and protein levels were confirmed by RT-qPCR and immunoblotting, respectively. Non-targeting siRNA (NC) was used as a control. It showed that si-RPS3 efficiently reduced RPS3 RNA and protein levels (Figure 5a,b). Next, we detected the HMGB1 levels in the supernatant harvested from NC or si-RPS3 transfected cells at 24 hpi. Compared to NC-transfected cells with PRRSV infection, silencing of RPS3 in PRRSV-infected cells led to considerably lower levels of HMGB1 in the supernatant (Figure 5c). This result indicates that RPS3 has a role in the HMGB1 secretion induced by PRRSV infection.

### 3.5. HMGB1 Phosphorylation at Threonine-51 Is Involved in HMGB1 and RPS3 Interaction

**Figure 6:**
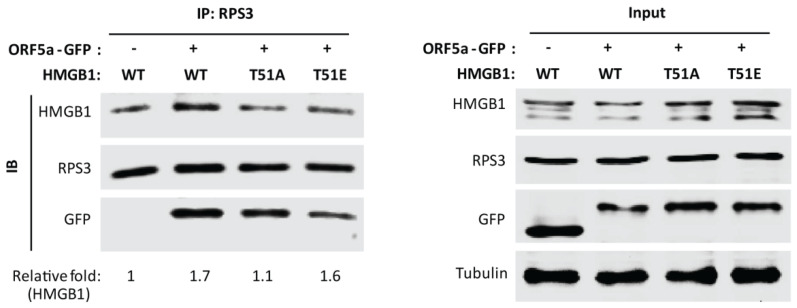
Phosphorylation of HMGB1 at Thr-51 residue is important for HMGB1 binding with RPS3. HEK293T cells were co-transfected with HMGB1 WT, T51A, or T51E plasmid and GFP or PRRSV ORF5a-GFP plasmid. At 30 h post-transfection, the cells were harvested for IP with RPS3 antibody, followed by IB. Relative levels of HMGB1 are shown as folds below the images after normalization with RPS3 in densitometry analysis. The inputs were also included in IB.

Since phosphorylation of HMGB1 at threonine-51 was associated with PRRSV induced HMGB1 secretion, we further determined whether the threonine-51 phosphorylation affected the interaction between HMGB1 and RPS3. HEK293T cells were co-transfected with PRRSV ORF5a-GFP plasmid and HMGB1 WT, HMGB1 T51A, or T51E plasmid. Co-IP with anti-RPS3 antibody was done, followed by immunoblotting of HMGB1. The result showed that in the cells with ORF5a expression, RPS3 precipitated less HMGB1 T51A than HMGB1 WT and HMGB1 T51E (Figure 6). The result suggests that RPS3 has a weaker interaction with HMGB1 T51A than HMGB1 WT and T51E, which indicates that HMGB1 phosphorylation at threonine-51 may enhance the interplay of HMGB1 and RPS3. Moreover, we discovered that ORF5a-GFP was also present in RPS3 precipitates (Figure 6), indicating their interaction.

### 3.6. PRRSV Increases RPS3 Expression and Nuclear Accumulation In Vivo

**Figure 7:**
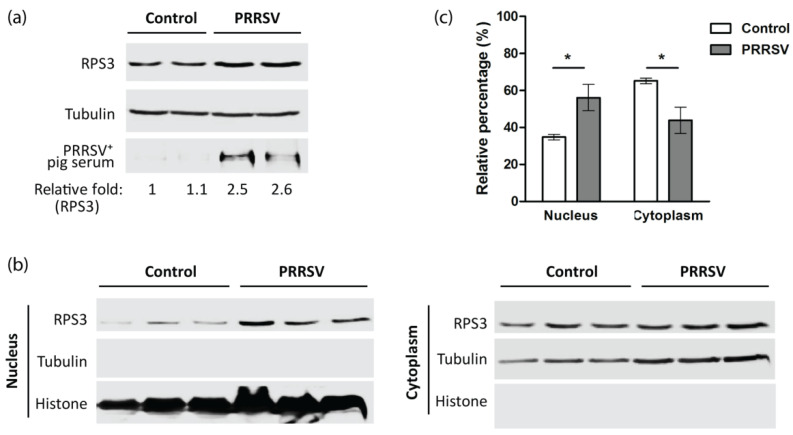
PRRSV infection promotes RPS3 expression and nuclear accumulation in PAMs. (**a**) PRRSV infection increases RPS3 expression. The pulmonary alveolar macrophages (PAMs) from PBS or PRRSV HuN4-inoculated pigs were subjected to IB. (**b**) PRRSV infection facilitates the nuclear accumulation of RPS3. PAMs were subjected to subcellular fractionation, followed by IB of RPS3. The same blot was probed with antibodies against tubulin and histone H2A as controls for loading and fractionation. (**c**) Densitometry analysis of the digital blotting images in (**b**). The RPS3 band intensity of each fraction is shown as the relative percentage of the summed density of both corresponding cytoplasmic and nuclear fractions after normalization with tubulin and histone for cytoplasmic and nuclear fractions, respectively. Significant differences between the control group and the PRRSV-infected group are denoted by “*” for *p* < 0.05.

To determine the effect of PRRSV on RPS3 expression, we detected PAMs that were collected from PRRSV or PBS-inoculated pigs [10]. In comparison with the PAMs of the mock-infected pigs, the PAMs from the PRRSV-infected pigs had a higher level of RPS3 (Figure 7a). As activated RPS3 is known to be present in the nucleus to play its extra-ribosomal functions [22,23], we conducted nucleocytoplasmic fractionation. The results showed that in PAMs from PRRSV-infected pigs, the elevated RPS3 was mainly distributed in the nucleus (Figure 7b). Densitometry analysis of the blotting results showed that in PAMs from control pigs, there was 34.8% of RPS3 in the nuclear fraction, whereas there was 56.2% of RPS3 in the nuclear fraction of PAMs from PRRSV-infected pigs (Figure 7c). This result demonstrated that PRRSV induced RPS3 elevation and nuclear accumulation. The above results suggested that PRRSV might induce HMGB1 secretion via promoting RPS3 expression, nuclear translocation, and direct interaction with HMGB1.

## 4. Discussion

Extracellular HMGB1 is a well-recognized pro-inflammatory mediator [9,29,30]. PRRSV induces HMGB1 secretion via activating PKCδ, a serine/threonine kinase, consequently exacerbating inflammatory damage [12]. Our results demonstrate that phosphorylation at threonine-51 residue within the NLS of HMGB1 is crucial for the HMGB1 secretion induced by PRRSV infection. PRRSV infection enhances the interaction between HMGB1 and RPS3, which is regulated by phosphorylation of HMGB1 at threonine-51. Moreover, PRRSV induces RPS3 elevation and nuclear accumulation in vivo.

HMGB1 is a ubiquitous non-histone chromatin-binding protein and is typically located in the nucleus [31,32]. Translocation from the nucleus to the cytoplasm is a critical step for its secretion, which is finely conditioned by post-translational modification, such as acetylation and phosphorylation [14,15,17,18,19,33,34]. In sepsis mice and lipopolysaccharide (LPS)-stimulated cells, activated JAK/STAT signaling promotes HMGB1 acetylation at lysine residues within the NLS, which results in HMGB1 cytoplasmic accumulation and release [18]. In cancer cells, HMGB1 phosphorylation at serine residues 35, 39, 42, 53 and 181 are critical for cytoplasmic localization of HMGB1 [20,21]. Besides, in the macrophages stimulated by tumor necrosis factor-α or LPS, the serine residues 35, 39, 42, 46, 53, and 181 are the major phosphorylation sites of HMGB1 involved in the cytoplasmic relocation and its eventual secretion [17,19]. Our previous study noted that serine/threonine kinase PKCδ activation is necessary for the HMGB1 secretion induced by PRRSV infection [12]. Therefore, we reasoned that HMGB1 phosphorylation is the inducement of its secretion in PRRSV-infected cells. In this study, we evaluated HMGB1 phosphorylation levels at serine or threonine residues and found that HMGB1 phosphorylation at threonine was greatly increased in PRRSV-infected cells. The result suggests that threonines might be the main targets for HMGB1 phosphorylation. By constructing and utilizing HMGB1 point mutants, we identified threonine-51 as the major phosphorylation site and that it is associated with the HMGB1 secretion induced by PRRSV infection.

PKC is the major kinase family catalyzing HMGB1 phosphorylation [35]. It has many isoenzymes classified into three subgroups: classical PKCs (cPKCs; α, β, and γ), novel PKCs (nPKCs; δ, ε, η, and θ), and atypical PKCs (aPKCs; ζ, ι, and λ) [36]. Oh et al. reported that in LPS-stimulated macrophages, HMGB1 is phosphorylated by cPKC in the nucleus, and that PKCα and PKCγ directly interact with HMGB1 in the nucleus [19]. In colon cancer cells, HMGB1 binds with many isoforms of PKC, including PKCδ, ζ, ι, and λ, in which PKCδ mainly binds with nuclear HMGB1, while others predominantly bind to cytoplasmic HMGB1 [21]. We previously discovered that PRRSV induces HMGB1 translocation and secretion via PKCδ activation and that PRRSV ORF2b and ORF5a proteins are responsible for the induction [12]. Therefore, in the present study, we tried to detect the interaction between HMGB1 and PKCδ in MARC-145 cells at 6, 15, and 24 h post PRRSV infection, as well as in HEK293T cells with PRRSV ORF2b or ORF5a expression. However, the co-IP results suggest that the HMGB1 interaction with PKCδ might be transient or beyond detection in this assay. It is also possible that there might be some other proteins involved in the HMGB1 phosphorylation. Indeed, MS analysis of the HMGB1 co-IP product identified RPS3.

RPS3 is a component of the 40S ribosome. Other than being involved in protein translation, RPS3 also has multiple extra-ribosomal functions, such as modulating DNA repair [37,38], gene transcription [27,39,40], apoptosis [41], and host-pathogen interactions. He et al. revealed that BfrB, an effector of *Mycobacterium tuberculosis*, subverts the host innate immune system by binding and reducing the nuclear abundance of RPS3 [42]. The virulence protein of Escherichia coli can also bind to RPS3 and blocks the transcription of nuclear factor-kappa B (NF-κB) target genes by attenuating nuclear translocation of RPS3, thereby promoting bacterial colonization and diarrhea [43,44,45]. In addition, RPS3 binds the 3′-untranslated region of the hepatitis C virus [46], and its expression level may be important to mouse resistance to the H5N1 influenza A virus [47]. In West Nile virus and yellow fever virus infection, RPS3 is involved in viral replication [48]. In this study, we found that PRRSV induced PRS3 elevation and nuclear accumulation in PAMs in vivo. The silencing of PRS3 efficiently suppressed HMGB1 secretion in cultured cells, which indicates that RPS3 is related to HMGB1 secretion. Moreover, HMGB1 was found to interact with RPS3, and mutation of threonine-51 in HMGB1 to the inactivated (unphosphorylated) state weakened the interaction.

Other studies also indicate that the interaction between PKC and HMGB1 is rapid and transient. Oh et al. observed that PKCα and PKCγ directly interact with HMGB1 in the nucleus at 20 min after LPS treatment [19]. However, the co-localization of PKC and HMGB1 in the nucleus was greatly reduced at 1 h after LPS stimulation and declined to baseline or lower at 2 h after stimulation. At 18 h after LPS treatment, the translocated HMGB1 was detected in the cytoplasm [19]. Interestingly, although a direct interaction of PKCδ and HMGB1 was not detected, in this study we discovered that PRRSV promoted HMGB1 interplay with RPS3, and PRRSV ORF5a bound them. The knockdown of RPS3 expression hinders the HMGB1 secretion induced by PRRSV infection in cultured cells. These results suggest that RPS3 might be a mediator involved in HMGB1 secretion. It is known that RPS3 can be phosphorylated by PKCδ [49,50], which leads to RPS3 activation and the translocation into the nucleus to play its extra-ribosomal functions [43,51,52]. We noticed that PRRSV infection not only promoted the interaction between HMGB1 and RPS3 but also enhanced the interplay between PKCδ and RPS3 (Figure S1). The data suggest that RPS3 may act as an intermediator to increase the probability of HMGB1 phosphorylation by PKCδ, thereby promoting HMGB1 cytoplasmic translocation and secretion.

In conclusion, in this study we discovered that phosphorylation at threonine-51 residue within the NLS of HMGB1 is crucial for the HMGB1 secretion induced by PRRSV infection. PRRSV infection facilitates HMGB1 binding with RPS3 to enhance HMGB1 secretion. Moreover, PRRSV induced RPS3 elevation and nuclear accumulation in vivo. These findings contribute to our understanding of the pathogenesis of PRRSV infection.

## Figures and Tables

**Table 1:** List of primers used in this study.

Primer ^a^	Sequences (5′-3′)	Target Gene
T51A-F	GTGGAAGGCCATGTCTGC	HMGB1-T51A
T51A-R	GCAGACATGGCCTTCCAC	HMGB1-T51A
T51E-F	GGTGGAAGGAGATGTCTG	HMGB1-T51E
T51E-R	CAGACATCTCCTTCCACC	HMGB1-T51E
RPL32-F	CAACTGGCCATCAGAGTCAC	RPL32
RPL32-R	GTGCACATGAGCTGCCTACT	RPL32
RPS3-F	GCTGAAGATGGCTACTCTGGAG	RPS3
RPS3-R	ACAGCAGTCAGTTCCCGAATCC	RPS3

^a^ F: forward primer, R: reverse primer.

**Table 2:** Brief result of mass spectrometry.

Accession	Description	Gene Symbol
A0A0D9S9M0	Uncharacterized protein	LOC103219323
A0A0D9SB94	Uncharacterized protein	LOC103237177
A0A0D9R6U8	60S ribosomal protein L29	RPL29
A0A0D9SC49	Uncharacterized protein	LOC103238803
A0A0D9SCL1	Histone cluster 1 H1 family member d	LOC103222006
A0A0D9R3U8	NUFIP2, FMR1 interacting protein 2	NUFIP2
A0A0D9RHW7	Polyadenylate-binding protein	PABPC1
A0A0D9S9I8	60S ribosomal protein L13	RPL13
A0A0D9S7U2	Splicing factor proline and glutamine rich	SFPQ
A0A0D9S7K7	Y-box binding protein 1	YBX1
**A0A0D9QV93**	**Ribosomal protein S3**	**RPS3**
**A0A0D9SBN8**	**Uncharacterized protein**	
A0A0D9S125	LSM12 homolog	LSM12
A0A0D9QXD5	G3BP stress granule assembly factor 2	G3BP2
A0A0D9SD09	Histone H4	LOC103247796; LOC103221971; LOC103222007
		LOC103221993; LOC103222008
**A0A0D9RXA3**	**Uncharacterized protein**	
A0A0D9REQ6	G3BP stress granule assembly factor 1	G3BP1
A0A0D9RDI7	Ribosomal protein L26	RPL26
A0A0D9QZA0	Cell cycle associated protein 1	CAPRIN1
A0A0D9RGJ5	Ribosomal protein S14	RPS14
A0A0D9RBS5	EWS RNA binding protein 1	EWSR1

Note: MARC-145 cells were infected with HuN4 at an MOI of 1 for 24 h and harvested for IP with HMGB1 antibody. The IP elution was subjected to SDS-PAGE gel running, and then the gels were cut and digested for mass spectrometry. The results of mass spectrometry were searched against the Chlorocebus sabaeus database (Proteome ID: UP000029965). Proteins with peptide-spectrum matching value (PSM) >1 were listed in the table. In comparison with the IP precipitate from mock-infected cells, IP product from the PRRSV-infected cells carries unique proteins, shown in red and bold.

## Data Availability

All data associated with this manuscript are available in the body of the paper and in the Supplementary Material.

## Supplementary Materials

The following supporting information can be downloaded at: https://www.mdpi.com/article/10.3390/v14051002/s1, Figure S1: PRRSV infection exacerbates the interplay between PKCδ and RPS3; Table S1: mass spectrometry raw data (IP precipitate from mock-infected cells); Table S2: mass spectrometry raw data (IP precipitate from HuN4-infected cells).
