# Supplementary material for: PRRSV Induces HMGB1 Phosphorylation at Threonine-51 Residue to Enhance Its Secretion
Source: Viruses. 2022 May 8;14(5):1002. doi: 10.3390/v14051002 (PMC9144045; doi:10.3390/v14051002)
Supplement: Supplementary file 1 [file viruses-14-01002-s001.zip › Figure S1_PRRSV infection exacerbates the interplay between PKCδ and RPS3.pdf]

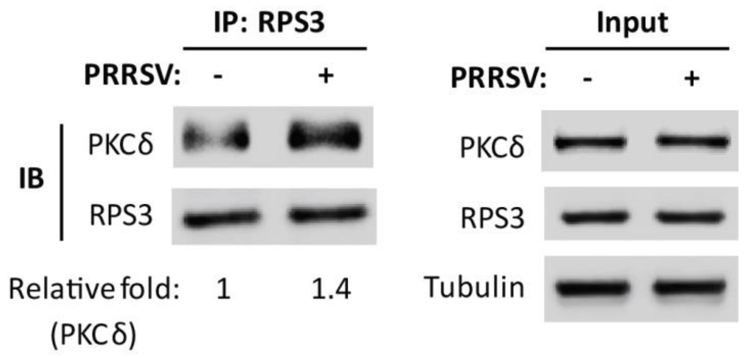

Figure S1. PRRSV infection exacerbates the interplay between PKCδ and RPS3. MARC-145 cells were infected with HuN4 at an MOI of 1 for 24 h and harvested cells for IP with RPS3 antibody, followed by IB with antibodies against PKCδ and RPS3. Relative level of PKCδ is shown as fold below the images after normalization with RPS3 in densitometry analysis. The input was included in IB.
